# belg: A Tool for Calculating Boltzmann Entropy of Landscape Gradients

**DOI:** 10.3390/e22090937

**Published:** 2020-08-26

**Authors:** Jakub Nowosad, Peichao Gao

**Affiliations:** 1 Institute of Geoecology and Geoinformation, Adam Mickiewicz University, Krygowskiego 10, 61-680 Poznan, Poland; nowosad.jakub@gmail.com; 2 State Key Laboratory of Earth Surface Processes and Resource Ecology, Beijing Normal University, Beijing 100875, China; 3 Faculty of Geographical Science, Beijing Normal University, Beijing 100875, China

**Keywords:** Boltzmann entropy, configurational entropy, landscape patterns

## Abstract

Entropy is a fundamental concept in thermodynamics that is important in many fields, including image processing, neurobiology, urban planning, and sustainability. As of recently, the application of Boltzmann entropy for landscape patterns was mostly limited to the conceptual discussion. However, in the last several years, a number of methods for calculating Boltzmann entropy for landscape mosaics and gradients were proposed. We developed an R package **belg** as an open source tool for calculating Boltzmann entropy of landscape gradients. The package contains functions to calculate relative and absolute Boltzmann entropy using the hierarchy-based and the aggregation-based methods. It also supports input raster with missing (NA) values, allowing for calculations on real data. In this study, we explain ideas behind implemented methods, describe the core functionality of the software, and present three examples of its use. The examples show the basic functions in this package, how to adjust Boltzmann entropy values for data with missing values, and how to use the **belg** package in larger workflows. We expect that the **belg** package will be a useful tool in the discussion of using entropy for a description of landscape patterns and facilitate a thermodynamic understanding of landscape dynamics.

## 1. Introduction

Entropy is a core concept in thermodynamics, which is a branch of physics, and explains “almost all known physical processes in the universe” [1]. This concept (widely referred to as thermodynamic entropy) was first described by the German physicist Rudolf Clausius in the 1850s to discuss the change of unavailable energy during a spontaneous process [2] then modeled by the Austrian physicist Ludwig Boltzmann [3] using the famous Boltzmann equation (hence the term Boltzmann entropy). Entropy has been widely used to express the most remarkable law of classical physics [4], the second law of thermodynamics, as follows, “the entropy of a closed system increases continuously and irrevocably toward a maximum” [5]. In addition to its fundamental role in physics, it has found applications in diverse fields such as sustainability (e.g., Gao et al. [6]), image processing (e.g., Sawant and Manoharan [7]), urban planning (e.g., Fistola and La Rocca [8]), and neurobiology (e.g., Blokh and Stambler [9]).

The application of entropy has also been explored and discussed in landscape ecology (e.g., Forman and Godron [10]; Jiang et al. [11]; Naveh [12]; O’Neill et al. [13]; Wu and Loucks [14]; Zurlini et al. [15]), for the following reason. Boltzmann entropy provides a statistical method to quantify unavailable energy based on the number of microstates in the macrostate of a thermodynamic system.

Accordingly, by specifying this number with a landscape pattern, one computes the Boltzmann entropy of a landscape pattern and establishes a relationship between the pattern and the energy of the landscape. Such a relationship allows for a deeper understanding of landscape dynamics based on thermodynamic insights, and it can be expected to “provide a theoretical context which could help clarify and unify a large portion of landscape ecology research” [16]. However, the computation of Boltzmann entropy remained a problem in landscape ecology for a long time because researchers have no idea on “how to specify and measure the macrostate/microstate relations” (Bailey [17], p. 151). Indeed, as confirmed by Vranken et al. [18], “no thermodynamic entropy quantification methods have been proposed” (p. 61).

As a result, the use of Boltzmann entropy in landscape ecology has long limited to conceptual discussion, with Shannon entropy being used as an alternative in practical applications (e.g., Rocchini et al. [19]; Díaz-Varela et al. [20]). Shannon entropy (i.e., information entropy) was proposed by the American mathematician Claude Shannon [21] to quantify the information content of a telegraph message and laid the foundation of information theory [22,23]. It has been widely considered as Boltzmann entropy in essence, and both entropies are used interchangeably (e.g., Lopez-Ruiz et al. [24]; Mohajeri et al. [25]). However, considerable criticisms are emerging about the equivalence between the two entropies. More recently, Vranken et al. [18] concluded that Shannon entropy is “merely a formal parallelism” (p. 54) to Boltzmann entropy. They further observed that almost all applications of Shannon entropy to landscape ecology—including spatial heterogeneity, the unpredictability of pattern dynamics and, and pattern scale dependence—can be questionable in terms of thermodynamic basis. Such observations have drawn much attention and described as “astounding” by leading ecologists [16].

Therefore, calls have been recently made for returning from Shannon entropy to Boltzmann entropy in spatial sciences [26,27] and landscape ecology in particular [16,18]. To apply Boltzmann entropy, the primary and most fundamental step is to compute the Boltzmann entropy of a landscape pattern. Note that this step is also the most difficult and had limited Boltzmann entropy to a conceptual level for centuries. Fortunately, this step has been taken in recent years. Specifically, methods have been developed for computing the Boltzmann entropy of a landscape pattern represented either using a patch-mosaic model [28,29,30,31] or a gradient model [32,33], according to a recent review [34]. However, these methods are much more complicated than that of Shannon entropy in terms of the amount of computation, and they are challenging to implement in practice [35]. Therefore, there is a need for software tools for conveniently computing the Boltzmann entropy of a landscape pattern.

This study aimed at presenting an R [36] package, **belg**, for conveniently computing the Boltzmann entropy of a landscape pattern represented using a gradient model, namely, a landscape gradient. The gradient model was focused for two reasons: First, the gradient model could be more universally [37] because it “subsumes the patch-mosaic model as a special case” (McGarigal and Cushman [38], p. 118). Second, software tools for the patch-mosaic model have been developed [35]. It is expected that our package **belg**, associated with existing tools, will make Boltzmann entropy easy-to-compute with all kinds of landscape patterns, facilitating a thermodynamic understanding of landscape dynamics for sustainable development.

## 2. Methods and Materials

### 2.1. Boltzmann Entropy for Landscape Gradients

As mentioned in the preceding section, Boltzmann entropy was modeled using the Boltzmann equation. For the sake of completeness, we will first briefly introduce this equation and then present an overview of the methods for its computation with a landscape gradient.

**Figure 1:**
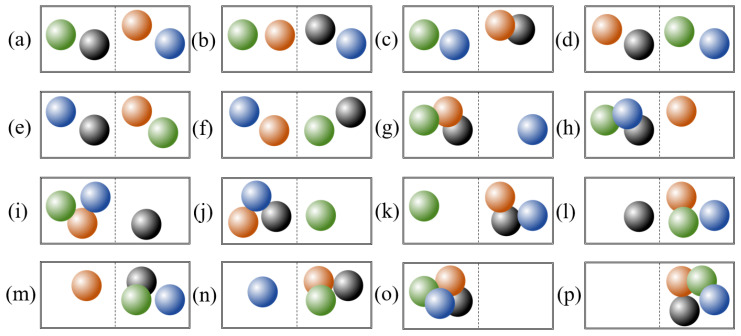
Microstates (**a**–**p**) of a closed container filled with four gas molecules. These microstates differ in the composition and configuration of molecules.

The Boltzmann equation involves two concepts: macrostate and microstate. The macrostate of a thermodynamic system is a state description of the system from a macroscopic perspective, using some easily measurable parameters (referred to as state functions) such as temperature, volume, and pressure [39]. By contrast, the microstate of a thermodynamic system is a state description from a microscopic perspective [40]. It is possible that many microstates are indistinguishable according to their macrostates [41]. In other words, a macrostate may correspond to many microstates. Based on these two concepts, the Boltzmann equation (*S*) is expressed as follows,

(1) $$S=k_{b}log\left(W\right)$$

where $k_{b}$ is the Boltzmann constant, and *W* is the number of microstates corresponding to the macrostate of a thermodynamic system. Let us take the classic example from thermodynamics: a closed container filled with four gas molecules, as shown in Figure 1a. This number of molecules serves as the macrostate of the container. If the position of each molecule is determined as either the left of the right half of the container, we can identify a total of sixteen microstates that share the same macrostate, as shown in Figure 1a–p. Therefore, the Boltzmann entropy of this container is according to Equation (1).

In dealing with a landscape gradient, researchers have developed two computational methods of Boltzmann entropy, namely, hierarchy-based (more precisely resampling-based) and aggregation-based. In addition, two new concepts have been introduced along with the computational methods: relative and absolute Boltzmann entropies.

#### 2.1.1. Hierarchy-Based Method for Computing Relative and Absolute Boltzmann Entropies

**Figure 2:**
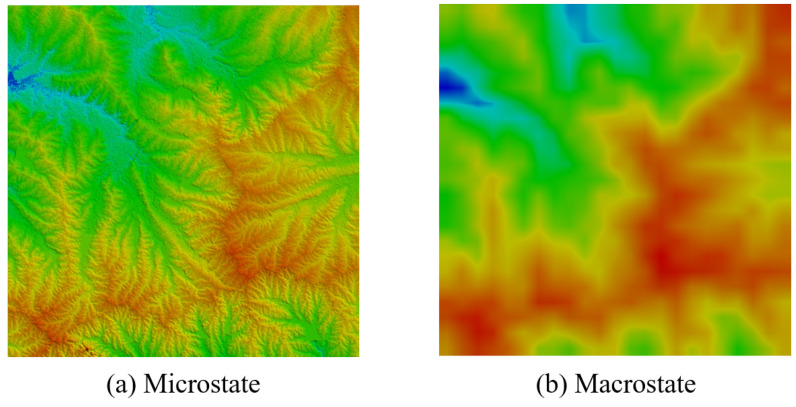
A pair of microstate and macrostate.

This method was the first effort towards computing the Boltzmann entropy of a landscape gradient. It was developed by Gao et al. [32] through rethinking the classic example of a closed container. Specifically, the macrostate of a landscape gradient was defined as an abstract (i.e., upscaled) version of the landscape gradient. Such a macrostate has a coarser resolution than the original landscape gradient, as illustrated in Figure 2. By contrast, the microstates corresponding to this macrostate was identified as the possible outcomes of the downscaling process from the macrostate to the original resolution, under the constraints that all outcomes share the same maximum, minimum, and average. The number of possible outcomes was substituted for *W* to compute Boltzmann entropy using Equation (1).

**Figure 3:**
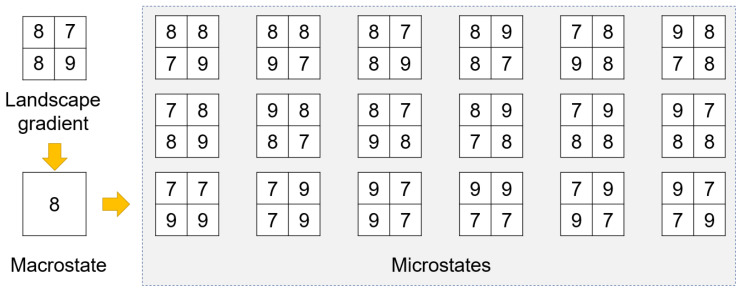
A simple landscape gradient, its macrostate, and all corresponding microstates.

To illustrate the preceding idea, let us take the simple example shown in Figure 3. In this example, the landscape gradient consists of only four cells (i.e., pixels), which are then upscaled to a single cell as the macrostate of the landscape gradient. This macrostate corresponds to a total of eighteen microstates, whose maximum, minimum, and average are all the same as the macrostate (i.e., 9, 7, and 8, respectively). Therefore, the Boltzmann entropy of this landscape gradient is $k_{b}log\left(18\right)$ according to Equation (1).

**Figure 4:**
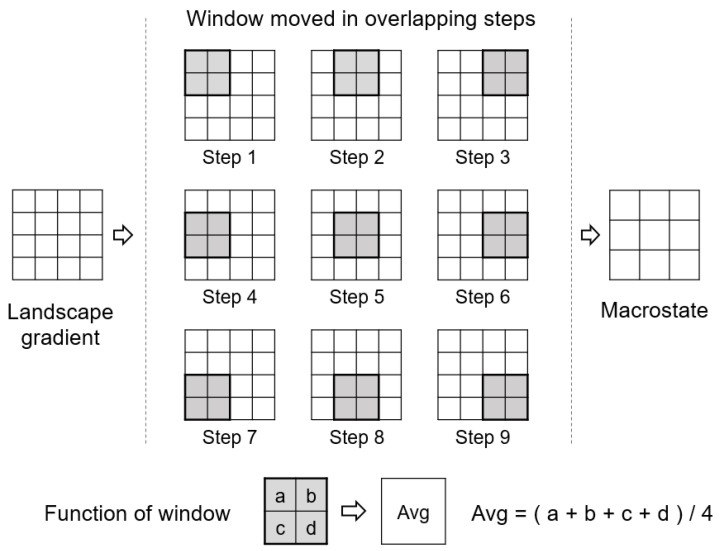
The resampling technique for generating the macrostate of a landscape gradient.

In dealing with landscape gradients with more than four cells, Gao et al. [32] proposed to employ a sliding window (i.e., moving window) of 2×2 cells to generate a macrostate, as shown in Figure 4. The sliding window moves in overlapping steps (i.e., one cell each time), generalizing a M×N landscape gradient to a (M−1)×(N−1) macrostate. Such a generalization technique has been referred to as resampling in multiscale spatial representation [27]. Then, the total number of microstates was calculated as the product of all the numbers of microstates determined with each window; in other words, the final Boltzmann entropy was computed as the sum of all the Boltzmann entropies of each window because the Boltzmann equation is a logarithmic function. Note that such a final Boltzmann entropy was referred to by Gao et al. [32] as relative Boltzmann entropy because it is a characterization of the landscape gradient in relative to its macrostate.

In addition to relative Boltzmann entropy, Gao et al. [32] introduced a concept of absolute Boltzmann entropy, which is the sum of (a) the relative Boltzmann entropy of a landscape gradient and (b) the relative Boltzmann entropies of all the up-scaled versions of the landscape gradient generated by iteratively applying the resampling technique. As the most generalized version (i.e., ultimately upscaled version) is a single pixel, whose relative Boltzmann entropy is zero, the following two statements are true. First, the absolute Boltzmann entropy of a landscape gradient has been calibrated to zero. Second, a comparison between two absolute Boltzmann entropies is meaningful because they have the same reference point. The relationship between relative and absolute Boltzmann entropies can be compared to that between relative and absolute heights or velocities.

#### 2.1.2. Aggregation-Based Method for Computing Relative and Absolute Boltzmann Entropies

**Figure 5:**
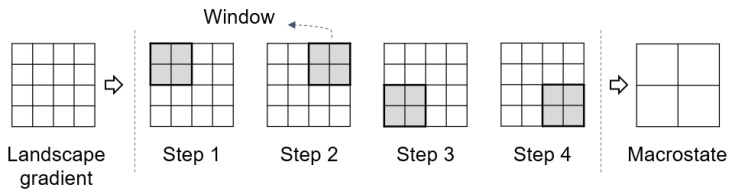
The aggregation technique for generating the macrostate of a landscape gradient.

**Figure 6:**
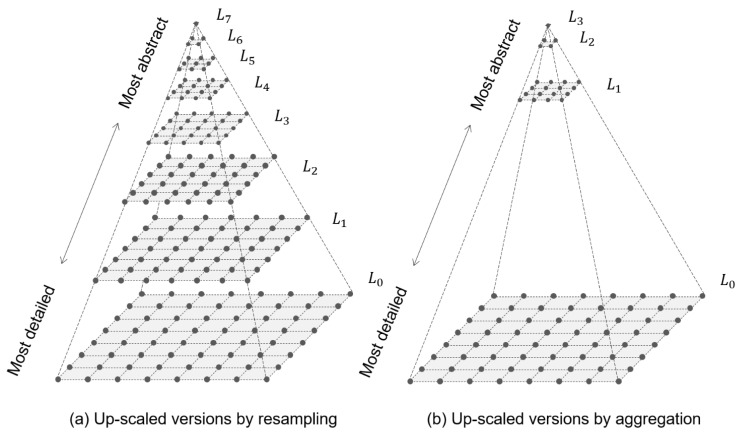
A comparisons between the hierarchical representation (upscaled versions) generated by iterative applying (**a**) the resampling technique and that by (**b**) the aggregation technique.

The second computational method [33] is similar to the first one. Their fundamental difference lies in the generalization technique. The new generalization technique is aggregation, where the sliding window is also of 2×2 size but moves in nonoverlapping steps (i.e., two cells each time), as shown in Figure 5. This difference results in two further changes. First, the size of the macrostate of a M×N landscape gradient has reduced from (M−1)×(N−1) to a (M/2)×(N/2). Second, the number of upscaled versions is less than that of the first computational method, as shown in Figure 6.

The second computational method outperformed the first one both in efficiency and effectiveness. The efficiency was improved because the amount of computation has been largely reduced. The effectiveness was improved in terms of thermodynamic consistency, which means whether the entropy computed using the Boltzmann equation is consistent with the second law of thermodynamics. Specifically, both the first and the second computational methods produce thermodynamically consistent relative Boltzmann entropies, but only the second method produce absolute Boltzmann entropies that are fully thermodynamically consistent.

### 2.2. Installation of the Belg Package

The *belg* R package is distributed under the MIT license and operates across operating platforms. It consists of two main parts. The first one is a user-friendly R interface, which allows for calculations of Boltzmann entropy on spatial data. The second part is a computationally fast C++ implementation of the underlining algorithms [42]. The latest stable version of the *belg* package is hosted on the CRAN repository and can be installed in R with the following code install.packages(“belg”). The development version is hosted on the GitHub platform at https://github.com/r-spatialecology/belg, and can be installed with remotes::install_github(“r-spatialecology/belg”). Additionally, this package also has a dedicated website at https://r-spatialecology.github.io/belg/ containing its documentation, installation instructions, and examples of use.

### 2.3. Core Functionality of the Belg Package

The *belg* package, in order to be used, must be first attached to an R session with library(belg). This package has one main function get_boltzmann(), which expects input data as the first argument and several additional arguments as the next arguments. Input data can be either a matrix, array, object from the *raster* package (RasterLayer, RasterStack, RasterBrick), or from the *stars* package [43,44]. The second argument, method, allows selecting a method of calculation used. It can be either “hierarchy” for the hierarchy-based method [32] or “aggregation” for the aggregation-based method [33]. The third argument, na_adjust, allows for scaling the output value based on the proportion of missing (NA) cells in the input data. Users can also decide on the used logarithm base with the base argument (“log”, “log2”, or “log10” (default)) and if they want to obtain relative or absolute entropy with the relative argument (TRUE or FALSE). The values returned by the get_boltzmann() function are unitless, as the Boltzmann constant $k_{b}$ is set to one [28].

## 3. Examples

### 3.1. Basic Example

The *belg* R package aims at calculating Boltzmann entropy values and it is well connected with existing R packages used to represent spatial raster data, including *raster* and *stars*. Except *belg*, the following examples also use *raster* to represent spatial raster data.


library(belg)

library(raster)


**Figure 7:**
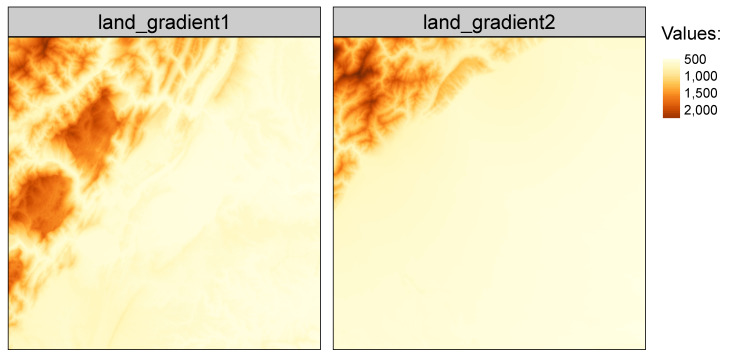
Two example landscapes: “land_gradient1” representing a more diverse landscape and “land_gradient2” representing a less diverse landscape.

The *belg* package has several build-in datasets allowing users to test its capabilities, including land_gradient1 and land_gradient2 (Figure 7). Both datasets have 512 rows and columns (262,144 cells in total), where the first one represents a more diverse landscape gradient than the second one.

The get_boltzmann() function calculates the Boltzmann entropy of landscape gradients. It requires, at least, one argument with input data to work. Other arguments are set by default. This function uses the aggregation-based method (method = “aggregation”), values are scaled based on the proportion of missing values (na_adjust = TRUE), a logarithm of base 10 is used (base = “log10”), and absolute entropy is calculated (relative = FALSE).


get_boltzmann(land_gradient1)

## [1] 188772.5

get_boltzmann(land_gradient2)

## [1] 121875.2


The above results confirm the visual evaluation—the values Boltzmann entropy of the first landscape is distinctly larger than of the second landscape.

### 3.2. Example with Missing Values

The calculations using the *belg* package can be extended to many landscapes. The data/sample_rasters folder has eight GeoTIFF files containing digital elevation models for different areas. Each file has 64 rows and columns and a resolution of 90 m.

All files can be found using the dir() function, and subsequently read to R using the lapply() and raster() functions.


sample_rasters_path = dir(“data/sample_rasters”, pattern =“.tif$”, full.names =TRUE)

sample_rasters = lapply(sample_rasters_path, raster)


The original methods for calculating Boltzmann entropy for landscape gradients by Gao et al. [32] and Gao and Li [33] works only on rasters without missing values. To solve this problem, we specified how to perform two steps of Boltzmann entropy calculations, upscaling and downscaling, in cases of data with missing values. In terms of upscaling, the average is computed using cells with values. When all values are missing, then the NA constant is returned. In downscaling, the number and positions of cells with missing values are preserved. More details about calculations for data with missing values are available in the package documentation at https://r-spatialecology.github.io/belg/articles/belg1.html.

**Figure 8:**
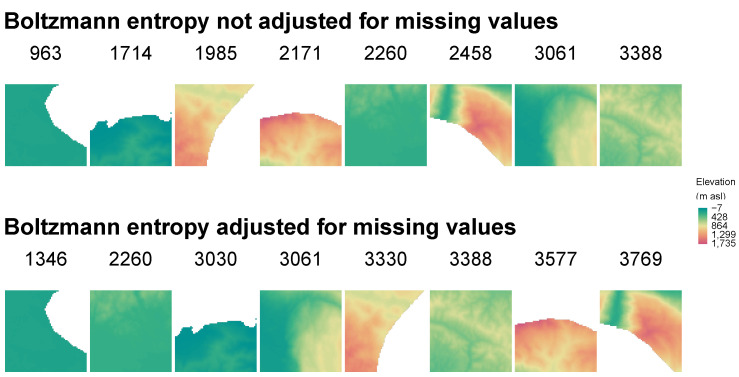
Examples of Boltzmann entropy values not adjusted and adjusted for missing values that were calculated for eight example local landscapes. Example local landscapes are sorted according to their Boltzmann entropy values.

This modification makes it possible to calculate Boltzmann entropy for data with different degrees of missing values. However, it makes the results dependable on the number of missing cells. For example, removing 20% of cells from a relatively uniform landscape will result in a decrease in Boltzmann entropy of about 20%. Therefore, it makes it impossible to compare landscapes with different proportions of missing values correctly. The top row in Figure 8 represents landscapes sorted by the values of Boltzmann entropy, calculated using the following code:



be_na = sapply(sample_rasters, get_boltzmann, na_adjust =FALSE)

be_na

## [1] 1713.9065 1985.3938 3061.0793 2457.6999 2259.5122 3387.7103 2171.1460

## [8] 963.3178


This approach returns larger values for landscapes without missing values. For example, the fifth landscape visually seems to be less complex than the fourth one, but it has more non-missing cells and therefore larger value of Boltzmann entropy.

To allow for proper comparison of landscapes with different levels of missing values, the *belg* package allows for adjusting the results:



be_na_adj = sapply(sample_rasters, get_boltzmann, na_adjust =TRUE)

be_na_adj

## [1] 3029.849 3330.128 3061.079 3768.903 2259.512 3387.710 3577.238 1345.754


When na_adjust is set to TRUE, then the initially calculated value of Boltzmann entropy is divided by the proportion (0–1) of cells without missing values. The adjusted values are presented in the bottom row in Figure 8.

### 3.3. Example of a Larger Workflow

**Figure 9:**
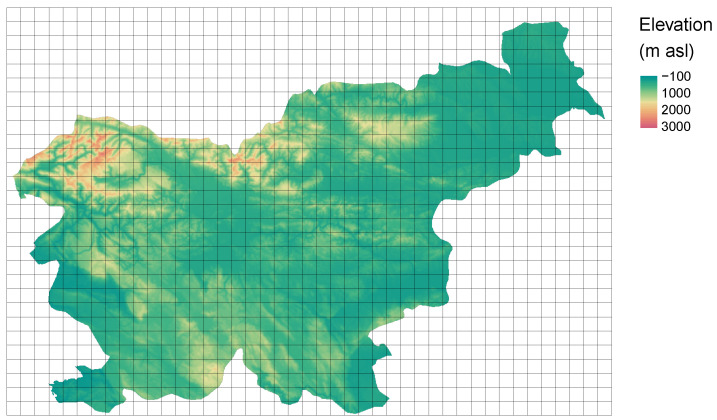
A grid with cells of 5760 by 5760 m imposed on the digital elevation model for Slovenia.

The svn_dem.tif contains a digital elevation model of 90 m resolution for the whole country of Slovenia (Figure 9).



svn_dem = raster(“data/svn_dem.tif”)


The R language [36] has extensive abilities for doing spatial data analyses, including data preparation, visualization, modeling, or communicating the results [45]. Therefore, it is possible to integrate the *belg* package into larger workflows. For example, users can create a polygonal grid using the *sf* package [46] and calculate Boltzmann entropy for a landscape in each grid cell.


library(sf)


The polygonal grid is created by extracting the bounding box of the elevation dataset, and specifying the new grid cell size in the st_make_grid() function.



svn_grid_geom = st_as_sfc(st_bbox(svn_dem))


svn_grid = st_make_grid(svn_grid_geom, cellsize =5760)


svn_grid = st_sf(id = seq_along(svn_grid),


         geom = svn_grid)


Next, the following code can be used to calculate Boltzmann entropy for each polygonal grid cell. The loop subsets a landscape for each polygonal grid cell, checks if it has any values other than NA, calculates entropy value, and returns it in a new column results.



svn_grid$results = NA

for (i in seq_len(nrow(svn_grid))){


  small_raster = crop(svn_dem, svn_grid[i, ])


  if(!all(is.na(getValues(small_raster)))){


   svn_grid$results[i] = get_boltzmann(small_raster)


  }


}


The output is a spatial object containing a column with calculated values of Boltzmann entropy.


head(svn_grid)


## Simple feature collection with 6 features and 2 fields


## geometry type: POLYGON


## dimension:   XY


## bbox:      xmin: 371601.3 ymin: 31015.3 xmax: 406161.3 ymax: 36775.3


## CRS:       +proj=tmerc +lat_0=0 +lon_0=15 +k=0.9999 +x_0=500000 +y_0=-5000000 ...


##  id               geom results


## 1  1 POLYGON ((371601.3 31015.3,... NA


## 2  2 POLYGON ((377361.3 31015.3,... NA


## 3  3 POLYGON ((383121.3 31015.3,... NA


## 4  4 POLYGON ((388881.3 31015.3,... 1139.992


## 5  5 POLYGON ((394641.3 31015.3,... 2834.936


## 6  6 POLYGON ((400401.3 31015.3,... 3120.389


**Figure 10:**
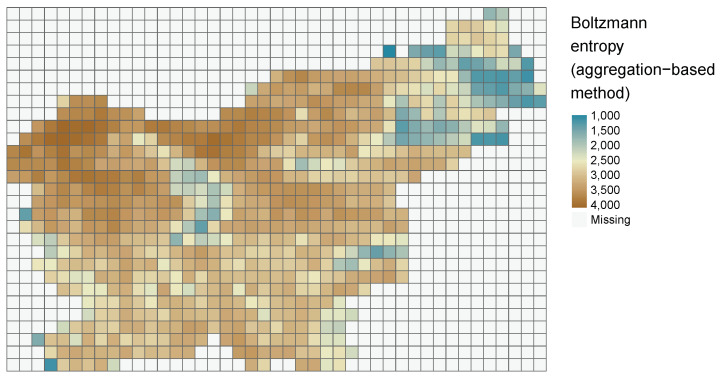
Values of Boltzmann entropy in a grid of 5760 by 5760 m calculated for the digital elevation model for Slovenia.

It can be visualized either using internal **sf** function plot() (plot(svn_grid["results"])) or external packages such as **tmap** [47] (Figure 10).

## 4. Discussion

In this paper, we introduced the **belg** R package for computing Boltzmann entropy of landscape gradients. It implements two computational methods—hierarchy-based and aggregation-based—of Boltzmann entropy using an efficient C++ code. An R interface allows for connecting methods in this package with an abundance of existing R packages for spatial data preparation or visualization. The **belg** package also expands the implemented methods by allowing calculations for rasters with missing values. We also presented three examples showing different aspects of the Boltzmann entropy calculations. Complete code and data to recreate all of the examples are available at https://github.com/Nowosad/belg-examples.

The **belg** package has a few limitations, however, they are mostly also the limitations of the implemented methods. It should be stressed that the absolute Boltzmann entropy calculated using the hierarchy-based method is not thermodynamically consistent [33], meaning that the entropy calculated using this approach does not increase continuously toward a maximum. The relative Boltzmann entropy is thermodynamically consistent, however, it does not allow for comparison between two different landscape gradients [32]. While the later proposed aggregation-based method is thermodynamically consistent, it only works on regular rasters with each dimension equal to *k* to base 2 [33].

The above limitations confirm that methods on how to derive Boltzmann entropy for spatial data are still an active area of research. Several concepts on how to compute the Boltzmann entropy on landscape patterns were proposed in recent years [28,29,30,31,32,33]. However, rarely the results of these methods were compiled and compared. Therefore, it is vital to have tools allowing to apply the previously mentioned methods on a diverse set of data. This not only could help to compare different methods underlining their strengths and limitations but above all, testing how well they represent lows of thermodynamics. Robust tools can also be used to evaluate relationships between proposed methods of computing Boltzmann entropy with existing measures based on Shannon entropy. It includes recently proposed conditional entropy, joint entropy, mutual information, and relative mutual information based on co-occurrence matrices [23].

Future improvements of the software will be aimed at implementing newly proposed methods for calculating Boltzmann entropy of landscape gradients. Additionally, while existing R packages, such as **parallel** and **future** [36,48], can be used together with **belg** to calculate Boltzmann entropy of many rasters in parallel, the package does not offer multi-core support for single raster images. Thus, it could be also worth adding parallel processing support for single large rasters. Finally, we look forward to the users’ comments and suggestions on potential changes and improvements in this package.
